# The Origin, Properties, Structure, Catalytic Mechanism, and Applications of Fucoidan-Degrading Enzymes

**DOI:** 10.3390/md23030097

**Published:** 2025-02-23

**Authors:** Yi Zhao, Limin Ning, Penghui Zhu, Jinju Jiang, Zhong Yao, Benwei Zhu

**Affiliations:** 1 College of Medicine, Nanjing University of Chinese Medicine, Nanjing 210023, China; zhaoyi@njtech.edu.cn; 2 College of Food Science and Light Industry, Nanjing Tech University, Nanjing 211816, China; zph18182591019@njtech.edu.cn (P.Z.); yaozhong@njtech.edu.cn (Z.Y.); 3 College of Food Engineering, Qingdao Institute of Technology, Qingdao 266300, China; jjjtianxie@163.com

**Keywords:** fucoidanase, fucoidan, structure, kinetic properties, substrate specificity, applications

## Abstract

Fucoidanase is a class of enzymes capable of hydrolyzing fucoidan, a complex sulfated polysaccharide found mainly in marine brown algae and some marine invertebrates. Fucoidan (FUC) has a wide range of potential health benefits and therapeutic effects, including antitumor, immunomodulatory, antiviral, and hypoglycemic activities. Fucoidanase can hydrolyze high-molecular-weight fucoidan into medium- and low-molecular-weight fucoidan. The low-molecular-weight fucoidan not only has good solubility, low viscosity, and high absorption rate but also retains the original biological activities of fucoidan. Fucoidanase has received much attention in recent years. This paper reviews the taxonomic origin, structure, enzymatic properties, and applications of fucoidanase to provide a reference for the study of fucoidanase.

## 1. Introduction

**Figure 1:**
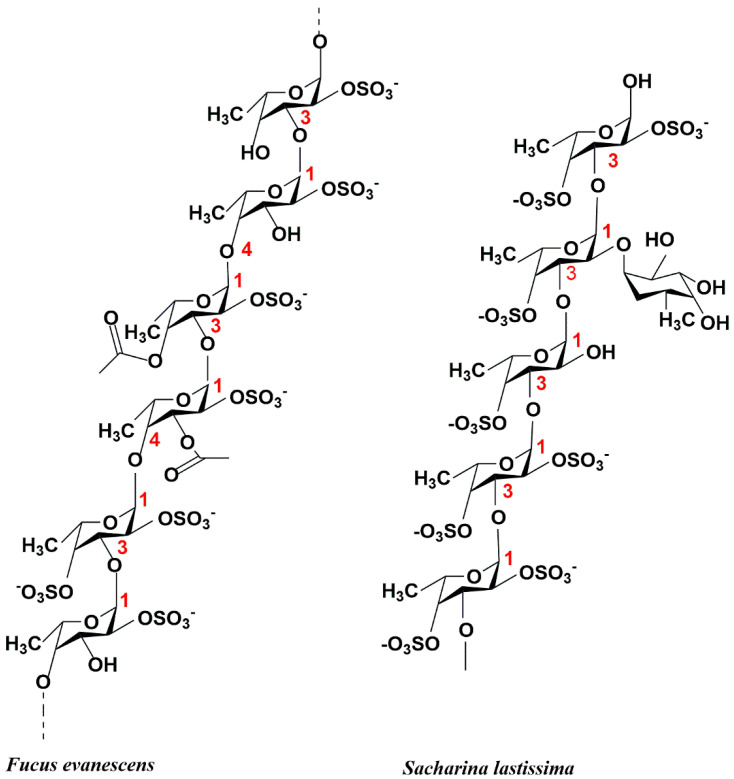
The structure of two different structural types of fucoidans.

**Figure 2:**
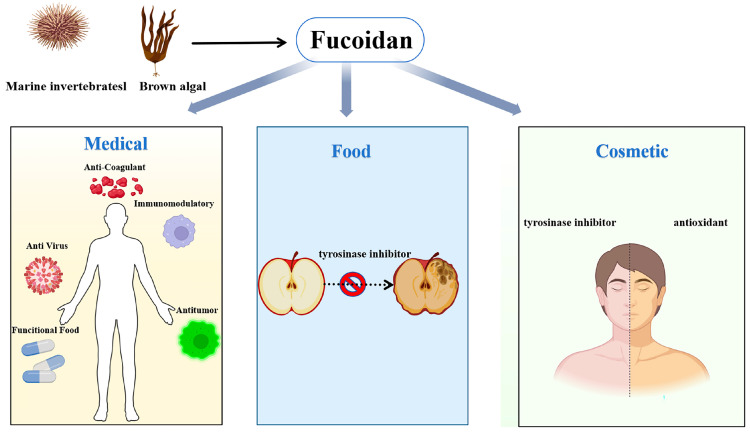
Biological activities of fucoidan.

Fucoidan (FUC), also known as sulfated fucoidan, is a heteropolysaccharide containing many L-fucose and sulfated groups. Fucoidan belongs to the family of sulfated homologous polysaccharides and heterologous polysaccharides, including polysaccharides mainly composed of sulfated fucose. Glucose, xylose, galactose, and mannose are also present in various fucoidans [1]. At present, the known fucoidan extraction methods include heat treatment method [2], acid treatment [3], microwave- and ultrasound-assisted extraction [4], photocatalysis [5], enzyme-assisted extraction (such as cellulase) [6], supercritical fluid method [7], and autohydrolysis (AH) [8]. Hot water extraction is the most commonly used method for extracting fucoidan in the laboratory. Fucoidan is a structurally diverse polysaccharide [9]. In general, the skeleton of fucoidan is mainly composed of two types: the first is composed of (1-3)-α-L-fucose as a repeating unit, and the second is composed of (1-3)/(1-4)-α-L-fucose as an alternating repeating unit (Figure 1). The research shows that fucoidan has antioxidant activity [10,11], which can effectively reduce inflammation caused by nervous system diseases and excessive free radicals [12]. It also has immunomodulatory activity [13], anti-inflammatory activity [14,15], anti-cancer activity [16], antibacterial [17], anti-coagulant [18,19], and other effects (Figure 2). It has been reported that the high molecular weight and high solubility of fucoidan limit its application in functional foods [20]. The structure and biological function of fucoidan are affected by many factors, such as raw material source, extraction method, and purification process [21].

The structure of fucoidan is related to species, extraction methods, and the season of algae extraction, which leads to heterogeneity of fucoidan, resulting in different chemical structures and compositions of fucoidan extracted from the same species [22]. The molecular weight of fucoidan is also heterogeneous [22]. The complexity of naturally occurring high-molecular-weight fucoidans is considered a major obstacle to therapeutic applications. Therefore, many studies have focused on producing low-molecular-weight fucoidan (LMWF) to easily elucidate its chemical structure and understand its pharmacological activity [23]. The important factors affecting the biological activity of fucoidan include molecular weight (MW), sulfate groups, sources, and extraction methods [24]. Compared with regular fucoidan, the biological activity and nutritional function of low-molecular-weight fucoidan are enhanced, which can be attributed to its lower molecular weight (MW) and greater exposure to sulfate groups [25,26,27,28,29]. Purity is an important parameter that affects the biological activity of fucoidan. For example, Gaurav Rajauria et al. [30] found that the antioxidant activity of purified fucoidan is lower than that of crude extract, which may be due to the presence of substances in the crude extract that can synergistically interact with fucoidan. Fucoidanase can hydrolyze macromolecular fucoidan into medium- and low-molecular-weight fucoidan. Low-molecular-weight fucoidan not only has good solubility, low viscosity, and high absorption rates, but it also retains a variety of the original biological activities of fucoidan [31]. It also has special biological functions, such as better immune regulation [32], anti-cancer [33], anti-oxidation [34], whitening [35], and other functions. Therefore, low-molecular-weight fucoidan has a wide range of applications and prospects in the pharmaceutical, nutritional and health products, cosmetics, and food industries. For example, low-molecular-weight fucoidan is beneficial in improving the disease control rate [36]. In addition, Ming Liu et al. [37] found that kelp fucoidan had no antibacterial activity, but its depolymerization product (<6 kDa) was rich in sulfate groups and had antibacterial activity. In addition, (FF5) fucoidan with a molecular weight of less than 5 kDa can be used as a promising browning inhibitor for fruit and vegetable preservation [38].

The structure and molecular weight of high-molecular-weight fucoidan exhibit heterogeneity; therefore, it is necessary to convert high-molecular-weight fucoidan into low-molecular-weight fucoidan [31]. Fucoidanases are a general term for a group of enzymes that specifically hydrolyze the glycosidic bonds in fucoidan. The different types of fucoidanase are classified into four families of glycoside hydrolases such as GH107 [39], GH168 [40], GH174 [41], and GH187 [42]. In particular, GH174 and GH187 are two families of fucoidanases that have been discovered and studied in recent years. For example, Marlene Vuillemin et al. [43] discovered and characterized a novel GH107 family of fucoidanase, thus further extending the function of GH107 family enzymes, which may pave the way for improved production of biologically active fucoidan oligosaccharides with potential for pharmaceutical applications; VTD Trang et al. isolated and characterized a novel fucoidanase, Fhf2Δ484, from the gut of the abalone *Haliotis gigantea* [44]. Therefore, fucoidanases are important for the production of biologically active fucoidan oligosaccharides. At the same time, fucoidanase can be used in the process of edible enzymes [45], cancer treatment [46], nutraceuticals [47], and deducing the chemical structure of complex fucoidan, so fucoidanases have a high value of application in the fields of food and medicine.

In recent years, with the growing market demand for functional foods and health products, the unique role of fucoidanase and the benefits of hydrolyzed fucoidan products have garnered increasing attention and research. Understanding the structural characteristics of fucoidanase is crucial for advancing our knowledge of its catalytic mechanism. With the development of structural analysis techniques, more fucoidanases have been characterized at the structural level. However, reports on the structure of fucoidanase remain limited, making it both necessary and beneficial to summarize and discuss the latest research findings. The structural study of enzymatic hydrolysis into fucoidan and the production of low-molecular-weight fucoidan provide indispensable tools. In the future, fucoidanase may be the main tool for targeted degradation of low-molecular-weight fucoidan. This paper reviews the progress in the structural characterization and enzymatic properties of fucoidanase, which may provide a theoretical basis for the subsequent application of low-molecular-weight fucoidan.

## 2. Sources and Classification of Fucoidanase

Fucoidanase is mainly derived from marine microorganisms, as shown in Table 1. Microorganisms, especially marine *Actinomycete* [46] and *Pseudomonas* [47], also have the ability to secrete fucoidanase. Researchers have successfully cloned and expressed fucoidanase genes in these microorganisms by genetic engineering, which improved the yield and activity of the enzyme. Plants, particularly brown algae like kelp [48] and *Sargassum* [49], are rich in fucoidanase, which plays a role in the metabolic processing of fucoidan. Certain marine invertebrates [50], such as Haliotis [43], also contain fucoidanase, which exhibits high activity and can be extracted from tissues like the digestive glands of these animals. Fucoidanase is named for its ability to break the glycosidic bonds between sulfated fucose units in the fucoidan molecule. Fucoidanases are a class of enzymes with specialized catalytic functions, which can be categorized into endo-fucoidanase, α-L-fucosidases, and sulfatases based on their modes of action. However, due to the highly complex molecular composition of fucoidan, these enzymes cannot be easily classified according to substrate specificity or the identified glycosidic bond sites.

These enzymes play an important role in living organisms, particularly in the production of low-molecular-weight fucoidan, where endo-fucoidanase is recognized as the best choice [51]. Endo-fucanases are glycoside hydrolases that catalyze the depolymerization of fucoidans by cleaving α(1 → 3)-linkages or α(1 → 4) linkage within the fucoidan backbone in an endo-acting manner. Based on sequence similarity, endo-α(1 → 4)-L-fucanases are classified within the glycoside hydrolase (GH) family 107, whereas endo-α(1 → 3)-L-fucanases are categorized into either family GH168 or GH107 or GH174 (www.cazy.org, accessed on 22 February 2025). Both enzyme families contain endo-fucoidanase that targets fucosyl bonds connecting sulfated α-L fucosyl residue [52]. For example, the study shows that the specific hydrolysis of α-(1,3) glycosidic bonds between 2-O-sulfated and non-sulfated fucose residues in the sulfated fucoidan from sea cucumber *Isostichopus badionotus* can be specifically hydrolyzed by Fun174A [41]. The specific enzymatic activity of Fun174A toward sulfated fucoidan molecules demonstrates its precise substrate specificity and underscores its potential as a valuable tool for targeted fucoidan hydrolysis. OUC-FaFcn1, a fucoidanase from *F. algicola*, is selective for the endolytic cleavage of α-(1,4) glycosidic bonds. It cleaves these bonds in fucoidan in a stoichiometric manner, primarily producing a disaccharide product [37]. In contrast, relatively few studies have focused on α-L-fucosidase. For example, the three fucoidanases isolated from Vibrio sp. N-5 by Furukawa et al. [53] were exonuclease-type enzymes, and their hydrolysis products consisted mainly of sulfated fucose and fucoidan. These enzymes play a crucial role in living organisms, particularly in the production of low-molecular-weight fucoidan, where endo-fucoidanases are considered the most effective choice [51].

The fucoidanase identified so far are classified into four families in the CAZy database (http://www.cazy.org/, accessed on 2 January 2025), including GH107 [39], GH168 [40], GH174 [41], and GH187 [42], with GH107 being the most extensively studied. For example, FcnA, the first fucoidanase of the GH107 family, was successfully isolated from the marine bacterium *M. fucanivorans* SW5T by Colin et al. [39] in 2006, while FunA, the first fucoidanase of the GH168 family, was extracted from the marine bacterium *Wenyingzhuangia fucanilytica* CZ1127 T by Shen et al. [40]. The first fucoidanase of the GH174 family, Fun174A, was successfully isolated from a marine bacterium by a research team of Liu et al. [41]. In 2024, Shen et al. [43] discovered the fucoidanase Fun187A in the marine bacterium *Wenyingzhuangia aestuarii* OF219, and the uniqueness of its homolog sequence marked the emergence of a new glycoside hydrolase family GH187. Some sources of fucoidanases have not been classified, such as the marine bacterium from *Vibrio* sp. N-2 [31], *Sphingomonas paucimobilis* PF-1 [54], and *Luteolibacter algae* H18 [55], marine fungi such as *Fusarium* sp. LD8 [56], *Dendryphiella arenararia* TM94 [57], *Dendryphiella arenaria* [58], and terrestrial *Aspergillus flavus* FS018 [59], and invertebrates such as marine mollusks *Lambis* sp. [60], *Patinopecten yessoensis* [61], and so on (as shown in Table 1).

**Table 1:** Classification and sources of fucoidanases.

Sources	Family	pH	OpT	K_m_	V_max_	M.W. (kDa)	Substrate Source	Specific Activity	Products	Ref.
*Muricauda eckloniae* (Mef1)	GH107	8	37 °C	ND	ND	45	*Fucus evanescens*		ND	[62]
*Muricauda eckloniae* (Mef2)	GH107	8	35 °C	ND	ND	105	*Fucus evanescens*	1.2 × 10^−3^ U_f_/μM	ND	[63]
*Formosa algae* strain KMM 3553 (FFA)	GH107	6.5–9.1	45 °C	ND	ND	96	ND	ND	ND	[64]
*Formosa algae* strain KMM 3553 T (FFA2)	GH107	6.5–9	25–37 °C	ND	ND	101.2	*Fucus evanescens*	ND	DP4, DP6	[65]
*Flavobacterium algicola* 12,076 (OUC-FaFcn1)	GH107	9.0	40 °C	ND	ND	110	Fucales	4.11 U/mg	DP2	[37]
*Alteromonas* sp. SN-1009 (tFda1B)	GH107	7.0	35 °C	3.88 ± 0.81 mg/mL	ND	100	*Kjellmaniella crassifolia*	0.0038 U/mg	ND	[45]
*Formosa haliotis* (Fhf1Δ470)	GH107	8	37–40 °C	ND	ND	71	*Fucus evanescens* *Fucus vesiculosus*	ND	DP4, DP8, DP10	[43]
*Formosa haliotis* (Fhf2Δ484)	GH107	8	37 °C	ND	ND	98	*Fucus evanescens* (*Fucus vesiculosus*, *Sargassum mccluei*, and *Sargassum polycystum*)	2.4 × 10^−4^ U_f_/μM	DP8, DP10	[44]
*Wenyingzhuangia fucanilytica* CZ1127 T (Fwf1)	GH107	6.4–7.2	24–35 °C	ND	ND	83	*Fucus evanescen*, *Fucus vesiculosus,* and *Sargassum horneri*	ND	DP4, DP6	[66]
*Wenyingzhuangia fucanilytica* CZ1127 T (Fwf2)	GH107	6.0–6.8	24–40 °C	ND	ND	95		ND	DP4, DP6, DP8	
*Psychromonas* sp. SW19D	GH107	ND	ND	ND	ND	ND	*Laminaria hyperborea* and *Macrocystis pyifera*	ND	ND	[67]
*Wenyingzhuangia fucanilytica* (FunA)	GH168	8.0	40 °C	1.05 ± 0.10 mg/mL	25.45 ± 0.97 U/mg	48	*Isostichopus badionotus*	13.7 U/mg	DP4	[40]
*Wenyingzhuangia fucanilytica* CZ1127 T (Fun168D)	GH168	7.5	35 °C	2.28 mg/mL	64.10 U/mg	49.5	*Isostichopus badionotus*	24.5 ± 1.1 U/mg	ND	[68]
							*Holothuria tubeulosa*	69.3 ± 0.9 U/mg		
*Wenyingzhuangia fucanilytica* CZ1127 T (Fwf5)	GH168	6.0–6.4	25–40 °C	ND	ND	44.3 ± 1	*Fucus evanescens*	ND	DP2, DP4	[69]
*Wenyingzhuangia fucanilytica* (Fun168E)	GH168	8.5	35 °C	1.07 mg/mL	5.07 U/mg	46.2	*Isostichopus badionotus*	ND	DP4	[70]
				3.66 mg/mL	4.46 U/mg		*Holothuria tubeulosa*			
*Wenyingzhuangia aestuarii* OF219 (Fun174A)	GH174	5.5	30 °C	5.60 mg/mL	11.04 U/mg	80	*Isostichopus badionotus*	2.87 U/mg	ND	[41]
ND (Fun174Sb)	GH174	7.5	35–50 °C	4.37 mg/mL	45.05 U/mg	54.3	*Isostichopus badionotus*	29.3 ± 2.1 U/mg	DP4	[71]
ND (Fun174Rm)	GH174	8.5	50 °C	2.84 mg/mL	4.27 U/mg	70.5		2.5 ± 0.1 U/mg	DP4	
ND (FunRi)	GH174	6.5	35 °C	1.18 mg/mL	11.05 U/mg	56.8		5.2 ± 0.1 U/mg	DP4	
*Wenyingzhuangia aestuarii* OF219 (Fun187A)	GH187	7.5	30 °C	3.51 mg/mL	1.51 U/mg	101	*Holothuria tubulosa*	1.4 U/mg	ND	[42]
*Cobetia amphilecti*	ND	8	30 °C	1.3 mg/mL	ND	35	ND	0.43 U/mg	ND	[51]
*Vasticardium flavum*	ND	3–4	ND	ND	ND	ND	*Stichopus variegatus*, *Holothuria spinifera*	ND	ND	[72]
*Sphingomonas paucimobilis* PF-1 (FNase S)	ND	6.0–7.0	40–50 °C	1.7 mg/mL	0.62 mg·min^−1^	130	ND	ND	ND	[54]
*Flavobacterium* sp. RC2-3 (Fcn1)	ND	8.0	50 °C	1.17 mg/mL	10.53 g/L·min	46.8	ND	332 U/mg	ND	[73]
*Fusarium* sp. LD8	ND	6	60 °C	ND	ND	64	ND	0.25 IU/mg	ND	[56]
*Aspergillus flavus* FS018	ND	5	55 °C	1.9 mg/mL	ND	ND	*Sargassum vulgare*	ND	ND	[59]
*Dendryphiella arenaria* TM94	ND	6	50 °C	6.56 mg/mL	ND	180	ND	0.32 IU/mg	ND	[56]

ND: not determined.

### 2.1. Structure of Fucoidanase

In the field of bioinformatics, predicting the structure of fucoidanase relies heavily on advanced computational tools and algorithms. Initially, researchers use sequence comparison techniques to align the amino acid sequence of the target fucoidanase with homologous proteins of a known structure. This comparison helps infer the three-dimensional structural features of the target enzyme [55,66,74]. Next, homology-based modeling methods are widely used to predict the structure of fucoidanase. Researchers select known structural proteins with high sequence similarity to the target enzyme as templates and construct a 3D model of the target enzyme through computer simulations. This process involves complex energy minimization calculations to ensure the stability and accuracy of the model [75]. Additionally, molecular dynamics simulations are used to further optimize and validate the predicted enzyme structure. By simulating the enzyme’s dynamic behavior under different environmental conditions, researchers can evaluate the model’s accuracy and the reliability of the functional predictions [76,77]. Finally, to verify the accuracy of the predicted results, experimental methods such as X-ray crystallography or nuclear magnetic resonance (NMR) spectroscopy are employed to determine the actual structure of fucoidanases [62,67,78]. Currently, the AlphaFold prediction algorithm (UniProt Registry Number: A0A1B1Y5R0) has been used for the GH168 family of fucoidanases [79]. In most cases, AlphaFold shows high accuracy in predicting protein folding. The final models were visually presented using PyMOL [44]. By comparing the predicted structures with experimental data, the prediction methods can be continuously adjusted and improved to enhance the accuracy and reliability of future predictions. At present, more and more fucoidanases have been excavated and characterized.

### 2.2. Three-Dimensional Structure of Fucoidanase

In recent years, with the development of structural biology techniques such as X-ray diffraction, more and more structural information of fucoidanase has been determined. At present, seven fucoidanases have been characterized. They are MfFcnA4 (PDB: 6DLH), MfFcnA9 (PDB: 6DNS), MfFcnA4_H294Q (PDB: 6DMS), P5AFcnA (PDB: 6M8N), Mef1 (PDB: 8BPD), FunA (PDB: 8YA77), and Fun168A (PDB: 8YA6) (Table 2).

**Figure 3:**
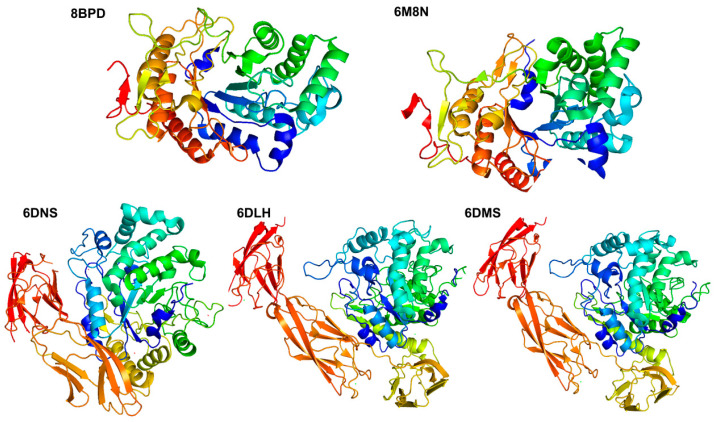
Crystal structures of characterized fucoidanase of the GH107 family (http://www.cazy.org/, accessed on 2 January 2025).

Most members of the GH107 family present a complex and variable structural domain structure, with only the (β/α) 8-barrel catalytic module (D1 structural domain) responsible for the conserved fucoidanase activity of the family member [80]. FcnA from Flavobacteriaceae was the first fucoidanase identified in the GH107 family, which consists of a 400 amino acid N-terminal structural domain, three repeating immunoglobulin-like folded structural domains, and a C-terminal structural domain of 80 amino acids. It is a modularly structured protein with a total length of 1007 amino acids and a molecular weight of approximately 110.3 kDa. This protein consists of several distinct structural domains, including a 28 amino acid signal peptide, a 390 amino acid N-terminal structural domain, three contiguous He-PIg structural domains (each containing approximately 105 amino acid residues), a signal peptide of 28 amino acids, an N-terminal structural domain of 390 amino acids, a C-terminal structural domain of 80 amino acids, and a C-terminal structural domain of 80 amino acids. The protein sequences are a 196-amino-acid region with low sequence similarity to known proteins and a 75-amino-acid C-terminal structural domain [39]. Together, these structural domains comprise the complex functions and properties of FcnA. Chelsea Vickers et al. [67] analyzed the structure of P5AFcnA (PDB: 6M8N) and P19DFcnA fucoidanase in the GH107 family. P5AFcnA reveals a single-domain organization containing the (β/α) 8-barrel domain. The (β/α) 8-barrel fold contains defects in the secondary structure, and the polypeptide region corresponding to one α-helix and one β-sheet does not form a regular secondary structure, which can be described as random coil. Three different truncations of MfFcnA, MfFcnA4_H294Q (6DMS), MfFcnA4 (PDB: 6DLH), and MfFcnA9 (PDB: 6DNS), were structurally characterized (Figure 3). Among them, MfFcnA4 has a single molecule in the asymmetric unit, which is a four-domain structure composed of a large N-terminal (β/α) 8-barrel domain. This N-terminal domain (D1) also contains the secondary structure defects mentioned above. In MfFcnA4, the D1 domain is connected to three consecutive Ig-like domains surrounding the D1 domain, and the three repeat domains after D1, called R1 to R3, have consistent folding with members of the immunoglobulin-like superfamily, and each domain binds to calcium atoms at the top. Two well-defined electron densities of metal ions were found in the N-terminal domain. One Ca^2+^ (Ca1) was identified as being bound to the loop leading to α-helix 5, coordinated in an octahedral arrangement via five oxygen ligands from the protein (side chains of Asp330, Asn335, and Asp336; the main-chain carbonyl group of Phe327 and Arg332) and a water molecule. A second Ca^2+^ (Ca2), potentially less strongly bound, was located near the C-terminus of α-helix. This ion is also octahedrally coordinated, comprising four water molecules and two protein oxygen ligands: one carboxylate from Asp79 and another from the main-chain carbonyl of Thr77 [67]. This suggests that Ca^2+^ promotes the activity of the enzyme. At the same time, the study indicates that the presence of a basic pocket in the −1 subsite can accommodate and complement the charge of the acidic sulfate ester group. This suggests that the enzyme may be capable of catalyzing the hydrolysis of sugars that contain sulfate groups, implying that the sulfation pattern of fucoidan could play a crucial role in its recognition by the enzyme.

Similarly, P5AFcnA also demonstrated the ability to accommodate the sulfate moiety through the basic patches in its active site groove. However, the unknown substrate structure of P5AFcnA and the differences in the surface profile of its active site relative to that of MfFcnA make the specific role of sulfate regulation in this enzyme less clear. Meanwhile, a truncated version based on the structure of MfFcnA4, which contains only the D1-R2 structural domain (amino acids Gln29-Asp623), was designed and named MfFcnA9. After prediction, the catalytic site residues of the GH107 fucoidan polymerase cover the conserved histidine (at position His276 in P5AFcnA) and aspartic acid (at position Asp201 in P5AFcnA), which act as acid–base catalysts and potential nucleophilic reagents, respectively.

Maria Dalgaard Mikkelsen et al. [62] found that the crystal structures of Mef1 and P5AFcnA have strong structural similarities. By sequence comparison analysis, the histidine and aspartate catalytic amino acids in Mef1 (PDB: 8BPD) are His270 and Asp187. In addition, there are four amino acids in the previously proposed -1 subsite, three of which are identified in Mef1 as Tyr128, Asn21, and Trp318. The 1.8 Å resolution crystal structure of Mef1 reveals a (β/α) 8-barrel structure with a structural architecture similar to that observed in the homologous D1 structural domains of the GH107-endonuclease P5AFcnA and MfFcnA (Figure 3). The active site of Mef1 is located in the center of the β-barrel and comprises the catalytic amino acids His270 and Asp187 as well as three conserved amino acids in the −1 subsite Tyr128, Asn215, and Trp318. There are two binding sites for Ca^2+^ ions in the crystal structure of Mef1: the Ca^2+^ ion (Ca1) is found in the well-organized Ca^2+^ site coordinated by Asp242, Asp246, and Asp248 and H_2_O bound to Asp240 via hydrogen bonding. The Ca1 site is further strengthened by hydrogen bonding from Tyr252 to Asp242 and from Ser282 to Tyr252 and is further stabilized by hydrogen bonding. The coordination of Ca^2+^ is facilitated by the backbone carbonyl groups of Ala136, Ser138, and Thr140, as well as the amide group of Asn142. The Ca^2+^ site is situated within the active site groove and is coordinated by two water molecules.

**Figure 4:**
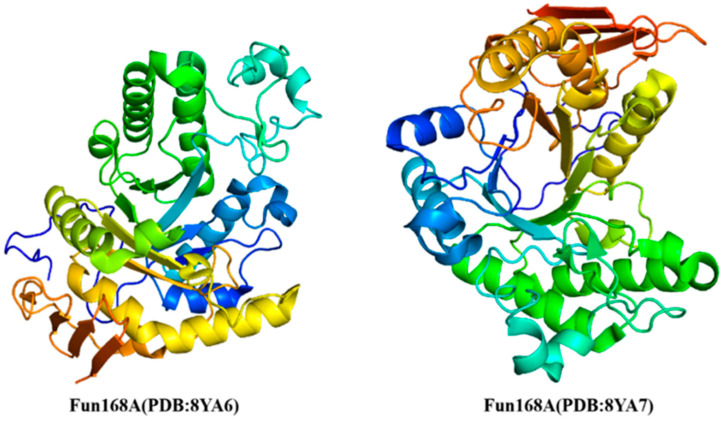
Crystal structures of characterized fucoidanases of the GH168 family (http://www.cazy.org/, accessed on 2 January 2025).

Chen et al. [69] reported the crystal structure of endo-1, 3-fucanase (Fun168A), and its complex with tetrasaccharide by X-ray diffraction technique for the first time. The crystal structure of Fun168A was determined at a resolution of 1.92 Å (PDB: 8YA6) (Figure 4). The asymmetric unit comprised a single protein copy, encompassing residues from Asp39 to Lys407. Fun168A predominantly adopted a canonical (β/α)_8_ triosephosphate isomerase (TIM) barrel fold (residues from Asp39 to Ala369), characterized by eight α-helices and eight parallel β-strands that alternate along the peptide backbone. Most of the α-helices consisted of over 10 amino acid residues, except for α5 and α8, which were composed of three residues each. Furthermore, a motif extending from Pro370 to Lys407 exhibited an antiparallel β-sheet fold consisting of three β-strands, with β9 tightly bound to α7 of the TIM barrel via a hydrophobic interface. The complex crystal of Fun168A with the tetrasaccharide product was obtained through soaking (PDB: 8YA7). Like the Fun168A-apo, the Fun168A-holo contained one molecule in an asymmetric unit. The substrate pocket of Fun168A was found in a groove at the barrel center, bordered by eight loops connecting the β-α segment. The positively charged pocket promoted binding to negatively charged substrates. The CAZy database (http://www.cazy.org/, accessed on 2 January 2025) contains the three-dimensional structure of the characterized fucoidanase (Figure 4).

### 2.3. Carbohydrate-Binding Modules (CBMs)

A carbohydrate-binding module (CBM), according to the CAZy database (http://www.cazy.org/, accessed on 2 January 2025) definition, is a contiguous amino acid sequence within a carbohydrate-active enzyme with a discreet fold having carbohydrate-binding activity. A few exceptions are CBMs in cellulosomal scaffoldin proteins and rare instances of independent putative CBMs. The requirement of CBMs existing as modules within larger enzymes sets this class of carbohydrate-binding protein apart from other non-catalytic sugar-binding proteins such as lectins and sugar transport proteins. Therefore, the binding ability of a CBM is usually matched with the activity of its parent enzyme [81]. CBMs are widely considered to be useful biotechnology tools for a variety of practical applications due to their finely tuned carbohydrate-binding capacity [82].

However, there have been few studies on the functional and structural characterization of the CBM domains in fucoidanase, and so far, only two carbohydrate-binding module (CBM) structures of fucoidanase have been reported. Notably, the discovery and cloning of a new CBM47 domain from a marine bacterium by Mei et al. represents significant progress in the study of carbohydrate-binding modules [83]. The carbohydrate microarray analysis described by Moller et al. [84] provides a method to assess the binding capacity of WfCBM47 and its specificity for sulfated fucose. The positive binding signal observed between WfCBM47 and Aj-FUC confirmed the ability of WfCBM47 to interact with sulfated fucose, thus validating its classification as a sulfated fucose-binding CBM.

In addition, this assay revealed that WfCBM47 did not bind to several other anionic polysaccharides, including chondroitin sulfate, dermatan sulfate, hyaluronic acid, and heparin. This underscores the specificity of WfCBM47 for sulfated laminar glycans and distinguishes it from other carbohydrate-binding modules. WfCBM47 is the first sulfated fucans-specific CBM. The expressed protein WfCBM47 shows specific binding ability to sulfated fucose with a backbone consisting of 1,3-α-fucose residues, highlighting its potential use in understanding and manipulating polysaccharide interactions. The second, recently, the gene encoding the fucoidanase Fun174A was identified from the marine bacterium *Wenyingzhuangia aestuarii* OF219, and this discovery led to the identification of a new structural domain with potential sulfated fucoidan binding activity [85]. AlphaFold2 was used to predict the structure of Fun174A and identify this unknown structural domain. Since this structural domain has a β-sandwich fold typical of carbohydrate-binding modules (CBMs), it has been proposed that this structural domain may represent a new family of CBMs. The discovery of Fun174A-CBM, a β-sandwich folded unknown structural domain in the fucoidanase Fun174A, was an important milestone in understanding the interaction of carbohydrate-binding proteins with sulfated fucoidan. Its ability to bind specifically to sulfated fucoidan was confirmed by biolayer interferometry, making it an important tool for the study of sulfated fucan-binding proteins. Quantitative analysis using BLI confirmed the binding ability of Fun174A-CBM to the Ib-FUC tetrasaccharide, thus validating its functionality and highlighting the role of BLI in quantifying carbohydrate-binding module interactions. The lack of similarity to known CBM sequences suggests that this is a new CBM family, expanding our understanding of CBM diversity and its role in identifying polysaccharides, particularly sulfated fucoidan. This identification offers great promise for advancing the study of sulfated fucan-binding proteins and contributes to our understanding of the physiological activities and potential applications of this important polysaccharide.

### 2.4. Enzymatic Properties of Fucoidanase

Due to the complexity of fucoidans structure, a single assay is typically insufficient to fully characterize the enzymatic activity of fucoidanase. To overcome this limitation, previous researchers have developed a range of assays for fucoidanase activity [52], incorporating factors such as changes in total or reducing sugar content [86,87] and variations in charge characteristics [88]. One major challenge with endo-fucoidanase is the absence of a universal and sensitive method for quantifying the reducing sugars released by endo-fucoidanase from different sources [45].

New FTIR-based assays have recently been developed for the quantitative measurement of endo-fucoidanase activity. This novel quantitative assay could pave the way for improved kinetic characterization and open new avenues for exploring endo-fucoidanase. The method relies on real-time spectral analysis of substrate and product changes during the enzyme’s action, using Fourier Transform Infrared Spectroscopy (FTIR) coupled with Parallel Factor Analysis (PARAFAC) [52]. Typically, the activities of fucoidanases are assessed through qualitative determination of their products. The biochemical characteristics of fucoidanases are closely linked to their source organism, structural features, and living environment.

The secondary structures of certain fucoidanases have been characterized. For example, the secondary structure composition of fucoidanase from *Fusarium* sp. LD8 was analyzed using FTIR, second-order derivative spectroscopy, and curve-fitting analysis of the amide I band. The results revealed that the β-sheet structure was dominant (58.6%), while the β-helix was the least abundant (12%). The α-turns and random coils accounted for 15.39% and 14.5%, respectively. The effect of temperature on the secondary structure was also examined. Below 60 °C, a decrease in β-turns and an increase in α-helices were observed in the amide I region. Above 60 °C, no significant changes were seen in the content of β-helices, β-sheets, random coils, or β-turns. The optimum reaction temperature for the enzyme was found to be 60 °C. Additionally, when the pH of the LD8 enzyme was either below or above 6.0, enzyme activity decreased rapidly, indicating that fucoidanase activity is closely related to the ratio of α-helix to β-helix structures [56].

As shown in Table 1, fucoidanases from marine bacteria exhibit maximum activity within the temperature range of 24–50 °C, while those from invertebrates and marine fungi show peak activity between 37–50 °C and 50–60 °C, respectively. However, some fucoidanases are more temperature-sensitive. For instance, fucoidanase from *Dendryphiella arenaria* TM94 has an optimal temperature of 50 °C but becomes semi-inactivated at 56 °C [57]. Similarly, fucoidanase from *Wenyingzhuangia aestuarii* has an optimal temperature of 30 °C and rapidly loses activity at temperatures above 35 °C [41]. The recently discovered fucoidanase from the GH187 family, Fun187A, also has an optimal temperature of 30 °C and remains stable after 24 h at 4 °C and 25 °C but rapidly loses activity at 40 °C [42]. It has been reported that the melting temperature (T_m_) of Mef2 is significantly influenced by the presence of Ca^2^⁺. In the presence of Ca^2^⁺, T_m_ increases from 38 °C to 44 °C. Ca^2^⁺ is present in the catalytic D1 structural domain of Mef2, and stripping the enzyme of Ca^2^⁺ using EDTA results in a complete loss of function [63]. The effect of Ca^2^⁺ on protein stability (T_m_) is evident, with T_m_ increasing by 6 °C in its presence, leading to a Tm of 44 °C compared to 38 °C in the absence of Ca^2+^ [63].

Some acidophilic, alkaliphilic, and thermophilic fucoidanases are listed in Table 1. Fucoidanases from marine bacteria generally exhibit maximum activity in neutral or slightly alkaline environments. For instance, the fucoidanase from *Fusarium algicola* (OUC-FaFcn1) has an optimal pH of 9.0 [37] and is sensitive to acidic conditions, making it the most alkali-tolerant fucoidanase discovered so far. Fucoidanases from invertebrates and marine fungi typically have an acidic optimal pH. The fucoidanase from the sea urchin *Strongylocentrotus nudus* exhibits an optimal pH of 3.0, characteristic of acidophilic enzymes [89]. Some enzymes, however, have an optimal pH in the alkaline range, such as the fucoidanase from the invertebrate *L. sitkana*, which has an optimal pH of 8.5 [88]. Additionally, the fucoidanase from the marine fungus Fusarium sp. LD8 has an optimal temperature of 60 °C, making it the most thermotolerant fucoidanase discovered to date

Meanwhile, metal ions have an activating or inhibiting effect on enzyme activity, where FFA1 from *F. algae* KMM 3553T is metal ion-dependent; FFA1 has activity only in the presence of Ca^2+^, Ba^2+^, Mg^2+^, and Mn^2+^ ions; the addition of EDTA accompanied by desalting of a medium completely inactivates FFA1 [90]; Ca^2+^ can promote the activity of most marine bacterial fucoidanase, such as Mef1, a Ca^2+^-dependent enzyme [62], but for the marine bacterium *S. paucimobilis*, *Alteromonas* sp. SN-1009 [45], and *Flavobacterium* sp. SW [91]; Zn^2+^ significantly reduced the activity of fucoidan from the *Alteromonas* sp. SN-1009 GH107 family of fucoidanase and was able to promote the activity of fucoidanase from *Formosa haliotis*; and Mn^2+^ increased the activity of tFda1B and Fhf1 but inhibited the activity of fucoidanase from *Wenyingzhuangia fucanilytica* CZ1127T [66]. It can be seen above that most fucoidanases are divalent cation-dependent enzymes, a conclusion that is confirmed by the crystal structures of fucoidanase since Ca^2^⁺ is found in the catalytic D1 structural domain [67]. In addition to the ability of metal ions to inhibit or activate fucoidanase, several substances can act as fucoidanase inhibitors or activators and thus affect their biological activity. Only two fucoidanase inhibitors have been reported. Silchenko et al. [92] were the first to identify an inhibitor of fucoidan hydrolase. Fucophloretol extracted from *Fucus evanescens* inhibited recombinant fucoidanase FFA from the marine bacterium *Formosan algae* KMM3553T and fucoidanase PPF from marine mollusks; the following year, this team found phenol (ND of 12–25) in *Fucus evanescens Costaria costata* metabolites, which could act as an inhibitor of fucoidanase from marine mollusks. Phenol (ND 12–25) may be part of the survival strategy of brown algae to protect themselves from marine herbivores and microbes [93].

## 3. Catalytic Mechanism and Substrate Specificity

**Figure 5:**
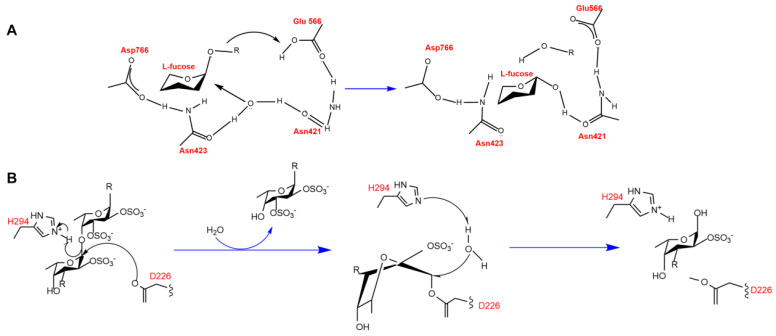
Schematic structure and catalytic active sites and catalytic mechanism of GH95 (**A**) 82 family fucosidases and GH107 (**B**) 66 family fucoidanase.

Fucoidanases bind to fucoidan substrates to form enzyme–substrate complexes that catalyze substrate degradation. A deeper understanding of the catalytic mechanism of fucoidanases can be achieved through in-depth analysis of the enzyme structure. There are few studies on the catalytic mechanism of fucoidanases. The crystal structure of AfcA,α-1,2-L-fucosidase from the GH95 family of *Bifidobacterium bifidum* (PDB: 2EAD) was elucidated by Nagae et al. [94]. The catalytic region of AfcA is an (a/a)_6_-helix barrel structure similar to that of phosphatases of bacterial origin. The catalytic region of AfcA is a (α/α)_6_ helical barrel structure and a calcium ion is present in AfcA to stabilize its conformation. AfcA degrades algal polysaccharides by a trans-conversion catalytic mechanism as a mono-substitution reaction (Figure 5). Glu566 acts as a proton donor for the atoms of the glycosidic bond, and Asp766-activated Asn423 and Asn421 form hydrogen bonds with water molecules, causing the water molecule to form a hydrogen bond with Asn423 and Asn421. Asp766-activated Asn423 and Asn421 form hydrogen bonds with water molecules, and the hydroxide ions formed by dissociation of the water molecules act as nucleophilic reagents to attack the heterocapital carbon of the fucoidan, thus opening the glycosidic bond, in which the distance between glutamate and asparagine is 10.5 Å, and this spatial distance allows for the interaction with the water molecules and the substrate simultaneously.

It has been reported that MfFcnA has endo-fucanase activity, and the active product pattern of P5AFcnA on fucoidan from *L. hyperborea* also suggests endo-hydrolysis activity. The structure of these enzymes reveals that the surface profile of the enzyme is the active site of the groove, which is consistent with the recognition and internal cleavage of the fucoidan chain. In addition, Chelsea Vickers et al. [67] also proposed that the different surface profiles of MfFcnA and P5AFcnA were consistent with the different specificity of fucoidanase from different sources through X-ray crystallography and nuclear magnetic resonance (NMR) studies of GH107 family fucoidanase, as well as in-depth comparison of BiAfcB active site residues in GH29 family enzymes. It was found that the endo-fucanase from the GH107 family showed structural and mechanism similarities with the α-l-fucosidase from GH29. The positional precision of Asp226 and Asp201 is sufficient to attack C1 in the fucose-1 subsite. In BiAfcB, where Glu217, which acts as an acid/base, is found to be in the MfFcnA4 and P5AFcnA were replaced by His294 and His276 in MfFcnA4 and P5AFcnA, respectively. The fact that the Ne2 nitro group of the histidine side chain was localized as acid/base suggests that His294 and His276 may have similar functions. Further determination of the crystal structure of MfFcnA4_H294Q revealed the specific catalytic role of His294 in MfFcnA and the similar role of His276 in P5AFcnA. Fucoidanase of the GH107 family shares the same structure and catalytic mechanism as that of fucoidanase of the GH29 family, but the GH107 unit has a very non-conservative active site structure, possibly reflecting the substrate specificity of GH107 family enzymes for degradation of fucoidan of different structures. The GH107 family uses a conserved catalytic mechanism to hydrolyze fucoidan, with the catalytic region being the classical TIM (β/α)_8_-barrel structure. Unlike the GH29 family of enzymes, the GH107 family replaces glutamate with histidine as the proton donor for the catalytic reaction (Figure 5).

The catalytic mechanism reported for glycoside hydrolases with transglycosylation activity is usually a retention mechanism because transglycosylation involves the transfer of a sugar group from one molecule to another while retaining its stereochemical structure. Glycoside hydrolases usually function by either a retention or inversion mechanism. Retention glycoside hydrolases have a two-step catalytic mechanism: glycosylation and deglycosylation. Transglycosylation products are produced when the receptor in the deglycosylation reaction is a molecule with a hydroxyl group rather than water. Recently, it has been found that FunA, a fucoidanase from the GH168 family, has transglycosylation activity, and it is hypothesized that this enzyme may have a catalytic retention mechanism [40].

In understanding the catalytic mechanism of fucoidanases, most researchers usually focus on the composition and structure of their active sites. The active site is where the catalytic reaction takes place and usually contains specific amino acid residues that interact with the substrate to facilitate the catalytic reaction (Table 3). Structural biology techniques, such as X-ray crystallography and nuclear magnetic resonance, allow for the resolution of the three-dimensional structure of the fucoidanase bound to the substrate, thus revealing the mode of action in the catalytic process. Among them, Tran et al. reported that Mef2 exhibits activity toward branched and unbranched fucoidan from *S. latissima* and *F. evanescens*, respectively, and is highly selective for fucoidan, not allowing galactose to be present in the oligosaccharide product, which is supported by NMR [63]. Mef2 is selective for fucose residues in the backbone and is selective for α (1,3) linkages only, but the active site region (probably located in the +2 and −2 subsites) will allow for branching of the fucose group. The amino acids in these subsites in fucoidanase have not been identified, as the crystal structures of substrate binding to the active site have not been disclosed. Only the active site and -1 subsite have been proposed. In the active site, a conserved amino acid in Mef2 has been changed from arginine to valine, whereas in the −1 subsite, a conserved asparagine, which is shown to be conserved in many other GH107 fucoidanases, is the α (1,3) chain-specific Fda1 and 2 as well as the serine in Mef2.

**Figure 6:**
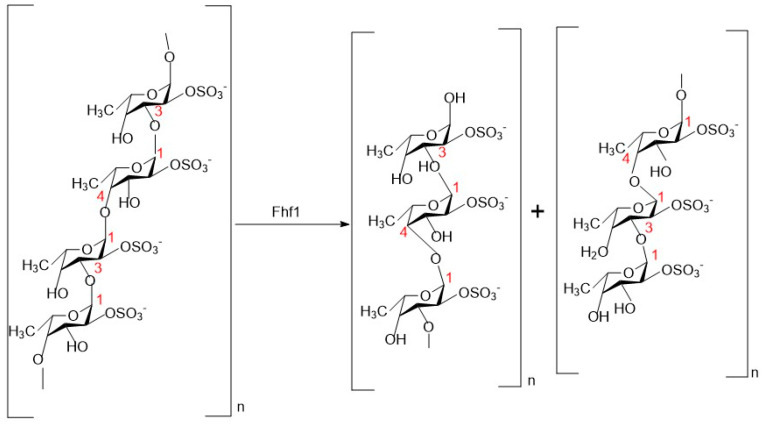
Site of action and products of the fucoidanase-Fhf1 [43].

Fucoidanase has specificity in recognizing and binding substrates, and its main function is to cleave glycosidic bonds in fucoidan molecules. The active sites of glycoside hydrolases are believed to comprise a series of sugar-binding subsites that recognize and accommodate specific structures within the substrate, facilitating subsequent cleavage [95]. These subsites can differ in quantity, arrangement, and specificity. The selectivity and spatial configuration of these sugar-binding subsites dictate the specificity of the glycoside hydrolases and the degree of polymerization (DP) of the resulting reaction products. However, it is worth noting that fucoidanase from different sources may differ in substrate specificity, which can affect the degradation of different fucoidan molecules. Members of the GH107 enzyme family are known to catalyze the hydrolysis of the α-1 → 4 or α-1 → 3-glycosidic bond between sulfated L-fucose residues in fucoidan and show specificity for certain modes of sulfation in fucoidan. In addition, the fucoidanase of GH174 and GH187 have been reported to cleave the α-1,3 glycosidic bond, and they belong to the group of endo-1,3-fucoidanase [71]. Some fucoidanase has been reported to be able to degrade the same fucoidan bonds. For example, Zueva et al. found that both FWf1 and FWf2 were able to catalyze fucoidan from groups of alternating 1 → 3- and 1 → 4-linked α-L-fucose residues with different sulfation modes by studying the kinetics of substrate hydrolysis, with FWf2 degrading fucoidan to tetra-, hexa-, and octa-saccharides [66]. Anastasiya et al. [96] investigated the substrate specificity of FWf3 and FWf4, novel fucoidanases belonging to the GH107 family. They effectively depolymerized fucoidan from *F. evanescens* and *S. horneri*, which have backbones consisting of alternating (1 → 3)- and (1 → 4)-linked sulfated α-L-glycosidic bonds. Both enzymes exhibited specificity for hydrolyzing the α-(1 → 4)-glycosidic bond between sulfated L-fucose residues. By analyzing the degradation products of the Fhf1 fucoidanase (Figure 6), Marlene Vuillemin et al. [43] found that this enzyme was able to degrade the α-(1 → 4)-glycosidic bond between 2-O-sulfated L-fucose residues in fucoidan from *F. evanescens*, and to produce tetrasaccharides, octasaccharides, and decasaccharides, and that fucoidanase that were able to degrade this glycosidic bond are FFA2, Fhf2 and FWf5 [28,41,97]. The endo-fucoidanase FcnA, FFA1, and FWf4 cleave fucoidan containing alternating 2-O- and 2, 3-di-O-sulfated L-fucose residues [98]. These enzymes are capable of generating oligosaccharides of varying degrees of polymerization. Both enzymes FunA (GH168) and Fun174A (GH174) catalyze the breakage of 1 → 3-glycosidic bonds between 2-O- and 2, 3-di-O-sulfated L-fucose residues of sulfated fucose. The 1 → 3-glycosidic bond break between sulfated and unsulfated L-fucose residues in sulfated fucose, and it was investigated that FunA and Fun174A did not show significant activity on fucoidan isolated from different species of brown algae [79].

However, some enzymes have cleavage specificity. For example, Shen et al. studied three endo-fucoidanases from the GH174 family and found that they exhibited novel cleavage specificities. Fun174Sb, Fun174Rm, and Fun174Ri were able to cleave the α(1 → 3)-bond between Fucp2S and Fucp2S [71], whereas the Mef1 endonuclease cleaved the α(1,4)-glycosidic bond between sulfated fucose residues on C255; the GH187 family of enzymes from Wenenberg, China, was also found to have cleavage specificity. α(1,4)-glycosidic bond between sulfated fucose residues [62], while FunA from *Wenyingzhuangia aestuarii* OF219 in the GH187 family cleaves the α-L-1,3-glycosidic bond between Fucp and Fucp (2OSO_3_^−^) [42]. These findings suggest that different species of fucoidanase exhibit complexity in degrading the fucoidanase exhibit complex specificities and activities, and these properties are essential for understanding their function in organisms and their potential role in industrial applications. Qiuet et al. [37] monitored the hydrolysis of fucoidan from the fucoidan genus *Murraya* by purifying the OUC-FaFcn1 enzyme using high-performance liquid chromatography (HPLC). OUC-FaFcn1 could degrade the fucoidan from the *Fucales genus*, whereas it hardly hydrolyzed the fucoidans from *L. japonica*, *I. badionotus*, *A. molpadioidea*, and *A. japonicus* with their backbones linked by α-1,3 glycoside bonds. Therefore, it can be speculated that OUC-FaFcn1 only acts on the α-1,4 glycoside bond of fucoidan because the backbone of Fucales genus-derived fucoidan is composed of α-1,3 and α-1,4 alternately linked L-fucopyranose residues. It is worth noting that research studies on substrate specificities of the GH107 family of fucoidanases are relatively few.

## 4. Application of Fucoidanase

### 4.1. Production of Low-Molecular-Weight Fucoidan

The fucoidanases are important tools for the production of low-molecular-weight fucoidan. Endo-fucoidanase catalyzes the specific hydrolysis of the α-L-fucan bond in fucoidan, which can be used to tailor fucoidan oligosaccharides and elucidate new structural details of fucoidan. Endo-fucoidanase catalyzes the specific hydrolysis of α-L-fucan bonds in fucoidan, which can be used to tailor fucoidan oligosaccharides and elucidate new structural details of fucoidan, as well as to adjust the biological activity of fucoidan. Studies have shown that the high molecular weight and solubility of fucoidan can limit its use in functional foods [20]. Fucoidan has a relatively complex structure, which varies with the species and the seasons [21,99,100,101], and therefore, the utilization of fucoidan is low. For example, Masura Honya et al. [100] extract crude fucoidan from kelp (*Laminaria japonica* Areschoug) cultivated in the southern part of Hokkaido Bay every month (from April to October). They found that the crude yield of fucoidan gradually increased from April to September, and the yield significantly increased after spore formation ended in October. On the same note, Bruhn et al. [21] also proposed that selecting the correct harvesting time can increase fucoidan production by 2 to 2.6 times. Fucoidanase can hydrolyze this large molecular fucoidan into medium- and low-molecular-weight fucoidans, providing a basis for the targeted preparation of specifically polymerized fucoidan oligosaccharide. For example, Y Qiu et al. [37] performed a product analysis of the fucoidanase OUC-FaFcn1 and found it to be the only biotechnological tool for the preparation of disaccharides from fucoidans. In addition, the GH174 family enzyme Fun174A, an endonuclease that may degrade Ib-FUC in a sustained manner, was found to be a favorable tool for the production of specific oligosaccharides from sulfated fucoidan [41]. Meanwhile, Fhf1 was able to break down fucoidan from *F. evanescens* into tetra-, octa-, and decasaccharides, and the structure of the products could be investigated by NMR spectroscopy [43]. Woo Jung Kim et al. [54] purified an enzyme (FNase S) that degraded Miyeokgui fucoidan (MF) from the marine bacterium *Sphingomonas paucimobilis* PF-1 to smaller fucoidan galactooligosaccharides (1000–4000 Da). This enzyme may be an important tool for structural analysis of fucoidan and the production of bioactive fucoidan. Meanwhile, fucoidan is also capable of preparing low-molecular-weight fucoidan with special biological activities. For example, Silchenko et al. [98] prepared fucoidan oligosaccharides using recombinantly expressed fucoidanase FFA1 and demonstrated that the fucoidan oligosaccharides have certain anti-cancer activities. Fucoidans are not only able to synthesize different low-molecular-weight fucoidans with ND, but also some low-molecular-weight fucoidans have special biological activities, which will provide important theoretical support for the research and development of functional drugs and foods in the future.

### 4.2. For Inferring the Structure of Fucoidan

The enzymatic depolymerization of fucoidan has attracted significant attention due to its ability to produce standardized fucoidan fragments. For example, in the presence of enzymes, fucoidan can be cleaved into low-molecular-weight products (LMPs) and high-molecular-weight polymeric components (HMPs) [98]. This HMP exhibits a regular structure composed of specific repetitive fragments. FunA, an enzyme with stringent specificity, is inactive against type II sulfated fucoidan but can be used as a biotechnology tool for structural studies of type I sulfated fucoidan. FunA also plays a role in facilitating the use of endo-1,3-fucosidases in bio-industrial applications, such as the specific production of α-1,3-linked sulfated fucoidan oligosaccharides [40]. Endo-1,3-fucosidases are crucial for both structural analyses of fucoidan and the preparation of fucoidan derivatives. However, the enzymatic properties and degradation products of these enzymes have not been extensively studied. The truncated endo-α(1 → 3)-fucoidanase Fda1 (tFda1B) from *Aeromonas alternata* was overexpressed and characterized. The degradation products of *Kjellmaniella crassifolia* fucoidan by tFda1B, analyzed by LC-ESI-MS/MS, confirmed that tFda1B belongs to the endo-α(1 → 3)-fucoidanase group and that the backbone of *K. crassifolia* fucoidan is composed of 1 → 3 fucoidan linkages. This enzyme can be utilized to elucidate the structure of fucoidan and may serve as a food enzyme [45]. At the same time, ongoing research is exploring ways to modify natural fucoidan to obtain more standardized and easily characterized derivatives. These studies are expected to provide new insights and methods for the development and application of fucoidan.

### 4.3. In Biotechnology

Fucoidanase has a wide range of applications in biotechnology. Firstly, these fucoidanases can be applied to the degradation of fucoidan, thereby providing energy and raw materials to organisms. In addition, fucoidanase can be used indirectly in the diagnosis and treatment of diseases. Fucoidan, as a key substrate, plays a crucial role in the conversion of brown macroalgae biomass into biofuels [58]. For example, Mohamed Gomaa et al. [58] found through research that the hydrolysis products of fucoidan and alginate would be a valuable resource for bioethanol production. Thus, fucoidanases are seen as potentially powerful tools for the production of biofuels. Secondly, fucoidanases are biocatalysts with promising applications and important roles in a variety of biotechnological fields [102]. Glycoside hydrolases with transglycosylation activity have been considered as potential biocatalysts for the large-scale synthesis of oligosaccharides or complex carbohydrates, and FunA, a member of the GH168 family, was reported to have transglycosylation activity, and it has a receptor for glycerol, methanol, and L-fucos [40].

### 4.4. Medical and Cosmetic Applications

Fucoidanase has a wide range of medical and cosmetic applications. For example, Manivasagan et al. [46] used *Streptomyces evanescens* to produce a novel fucoidanase through which gold nanoparticles were synthesized and found that these biosynthesized gold nanoparticles exhibited dose-dependent cytotoxicity against Hela cells, which opens up the possibility of novel fucoidanase for use in anti-cancer therapy. In addition, an endo-fucoidanase from *F. evanescens* was able to hydrolyze fucoidan to produce low-molecular-weight fucoidan, which is capable of altering biological activity during bone regeneration [103]. The use of fucoidan produced by a marine bacterial strain, *Wenyingzhuangia fucanilytica* CZ1127 T, allowed for the preparation of a series of fucoidan with different molecular weights (MWs), which were found to have a high degree of cytotoxicity and were able to be used in the treatment of cancer. A series of low-molecular-weight fucoidans with different molecular weights (MWs) were produced, which can be effectively utilized as natural antioxidants and are effective in preventing ethanol-induced gastric ulcers [97]. Fucoidanase also has a wide range of applications in the field of makeup. Fucoidan has been used to develop whitening cosmetics based on the principle of inhibiting tyrosinase activity by the bioactive substances produced by degrading fucoidan. It can reduce the production of dopa tyrosine, thereby inhibiting the formation of melanin and achieving skin-whitening effects [28].

### 4.5. Food Industry

Fucoidanase has a wide range of potential applications in the food industry. This fucoidanase can be used to degrade fucoidan in foods and improve the quality and taste of foods. For example, the addition of fucoidanase to foods such as noodles and steamed buns can improve the taste and digestibility of the food. In the past decades, sea cucumbers have attracted much attention due to their rich physiological activities. However, its species differentiation has not been investigated, and different fucoidanase can differentiate sea cucumbers based on the recognition of specific fucoidan [104]; meanwhile, FF5 prepared by degradation of fucoidanase from the *Flavobacteriaceae* sp. RC2-3, in terms of competitive inhibition, demonstrated that it was able to significantly slow down the browning and dehydration process of freshly cut apple slices through the inhibition of tyrosinase activity. Thus, fucoidanases may have potential applications in food processing and preservation, especially playing an important role in retarding the oxidation of fruits and maintaining their quality [38].

## 5. Prospects and Conclusions

Given the important role of fucoidanase in the development of fucoidan resources, the discovery of novel fucoidanase has become an important direction for future research. This requires us to continuously broaden our research horizons, strengthen interdisciplinary cooperation, make full use of modern biotechnological means, and deeply explore the enzyme resources in marine microbial resources. At the same time, we also need to strengthen the research on the mechanism of enzyme catalysis and the relationship between structure and function to provide a solid theoretical foundation for the development of new enzyme preparations.

In summary, as a natural substance with a wide range of biological activities, low-molecular-weight fucoidan has great potential for development and application. Enzymatic preparation, as the most promising extraction method for the future, will enable the production of more high-quality, high-activity fucoidan products. In this process, fucoidanase, as an indispensable tool and mediator, will play a crucial role. Therefore, enhancing research on fucoidanase will not only help us better understand and utilize this valuable marine resource but also make significant contributions to human health and sustainable development.

## Figures and Tables

**Table 2:** Structural characterization of fucoidanases.

Family	Enzyme	GeneBank	3D Structure Status	Resolution (Å)	PDB	Ref.
GH107	Mef1	KQC28683.1	(β/α) 8 barrel	1.8	8BPD[A]	[62]
	MfFcnA4	CAI47003.1Q08I46	(β/α) 8 barrel	2.20	6DLH[A]	[67]
	MfFcnA4_H294Q			2.85	6DMS[A]	
	MfFcnA9			2.24	6DNS[A]	
	P5AFcnA	AYF59291.1A0A452CSY7	(β/α) 8 barrel	1.55	6M8N[A]	
GH168	Fun168A	ANW96599.1 WP_068826898.1	(β/α) 8 barrel	1.92	8YA6[A]	[69]
	FunA			1.99	8YA7[A]	
	Poly41_55130	TWU32535.1	ND	1.7	9JOS	https://www.cazy.org/
				2.02	9JP2	
				1.4	9JP3	
	FUN168E	ANW96379.1		2.04	9JOM	
				1.97	9JOO	
	FUN168D	ANW96381.1		1.29	9JOC	
				1.5	9JOF	
				1.4	9JOG	
				1.36	9JOH	

ND: not determined.

**Table 3:** Catalytic bases of different fucoidanases.

Enzyme	Family	Action	Catalytic Bases	Active Site	Ref.
Mef2	GH107	endo-α-1,3-L-fucanase	Asp182/His260	ND	[63]
OUC-FaFcn1	GH107	endo-α-1,4-L-fucoidanase	Asp231	ND	[37]
tFda1B	GH107	endo-α(1,3)-fucoidanase	Asp202	ND	[45]
FWf1	GH107	endo-α-1,4-L-fucoidanase	Asp226/His294	ND	[66]
FWf2	GH107	endo-α-1,4-L-fucoidanase	Asp464/His537	ND	
FWf3	GH107	endo-α-1,4-L-fucanase	Asp401/His469	ND	
FWf4	GH107	endo-α-1,4-L-fucanase	Asp229/His297	ND	
Fhf1	GH107	endo-α-(1,4)-fucoidanase	Asp225/His293	ND	[43]
Mef1	GH107	endo-a(1,4)-fucoidanase	Asp187/His27	ND	[62]
Fun168E	GH168	endo-1,3-fucanase	ND	ND	[70]
FunA	GH168	endo-1,3-fucanase	ND	D206, E264	[40]
Fun174A	GH174	endo-1,3-fucanase	ND	D119, E120, E218	[41]
Fun187A	GH187	endo-α-1,3-L-fucanase	ND	ND	[42]

ND: not determined.

## Data Availability

Not applicable.

## References

[1] Oliveira C., Neves N.M., Reis R.L., Martins A., Silva T.H. (2020). A review on fucoidan antitumor strategies: From a biological active agent to a structural component of fucoidan-based systems. Carbohydr. Polym..

[2] Bird C.J., Ragan M.A. (2012). Eleventh International Seaweed Symposium. Proceedings of the Eleventh International Seaweed Symposium.

[3] Hahn T., Lang S., Ulber R., Muffler K. (2012). Novel procedures for the extraction of fucoidan from brown algae. Process Biochem..

[4] Rodriguez-Jasso R.M., Mussatto S.I., Pastrana L., Aguilar C.N., Teixeira J.A. (2011). Microwave-assisted extraction of sulfated polysaccharides (fucoidan) from brown seaweed. Carbohydr. Polym..

[5] Qi Y., Wang L., You Y., Sun X., Wen C., Fu Y., Song S. (2022). Preparation of low-molecular-weight fucoidan with anticoagulant activity by photocatalytic degradation method. Foods.

[6] Jayawardena T.U., Fernando I.P.S., Lee W.W., Sanjeewa K.K.A., Kim H.-S., Lee D.-S., Jeon Y.-J. (2019). Isolation and purification of fucoidan fraction in *Turbinaria ornata* from the Maldives; Inflammation inhibitory potential under LPS stimulated conditions in in-vitro and in-vivo models. Int. J. Biol. Macromol..

[7] Men’shova R.V., Lepeshkin F.D., Ermakova S.P., Pokrovskii O.I., Zvyagintseva T.N. (2013). Effect of pretreatment conditions of brown algae by supercritical fluids on yield and structural characteristics of fucoidans. Chem. Nat. Compd..

[8] Rodríguez-Jasso R.M., Mussatto S.I., Pastrana L., Aguilar C.N., Teixeira J.A. (2013). Extraction of sulfated polysaccharides by autohydrolysis of brown seaweed *Fucus vesiculosus*. J. Appl. Phycol..

[9] Ha V.T.N. (2022). Kinetics and Technology Functionality of Microbial Fucoidanase. Ph.D. Thesis.

[10] Rocha de Souza M.C., Marques C.T., Guerra Dore C.M., Ferreira da Silva F.R., Oliveira Rocha H.A., Leite E.L. (2007). Antioxidant activities of sulfated polysaccharides from brown and red seaweeds. J. Appl. Phycol..

[11] Koh H.S.A., Lu J., Zhou W. (2019). Structure characterization and antioxidant activity of fucoidan isolated from *Undaria pinnatifida* grown in New Zealand. Carbohydr. Polym..

[12] Lim S.J., Wan Aida W.M., Maskat M.Y., Mamot S., Ropien J., Mazita Mohd D. (2014). Isolation and antioxidant capacity of fucoidan from selected Malaysian seaweeds. Food Hydrocoll..

[13] Itoh H., Amano H., Kakinuma M., Noda H. (2002). Antitumor activities and immunological studies with algal polysaccharides. Fish. Sci..

[14] Lee S.-H., Ko C.-I., Ahn G., You S., Kim J.-S., Heu M.S., Kim J., Jee Y., Jeon Y.-J. (2012). Molecular characteristics and anti-inflammatory activity of the fucoidan extracted from *Ecklonia cava*. Carbohydr. Polym..

[15] Apostolova E., Lukova P., Baldzhieva A., Katsarov P., Nikolova M., Iliev I., Peychev L., Trica B., Oancea F., Delattre C. (2020). Immunomodulatory and anti-inflammatory effects of fucoidan: A review. Polymers.

[16] Senthilkumar K., Manivasagan P., Venkatesan J., Kim S.-K. (2013). Brown seaweed fucoidan: Biological activity and apoptosis, growth signaling mechanism in cancer. Int. J. Biol. Macromol..

[17] Shibata H., Kimura-Takagi I., Nagaoka M., Hashimoto S., Sawada H., Ueyama S., Yokokura T. (1999). Inhibitory effect of Cladosiphon fucoidan on the adhesion of Helicobacter pylori to human gastric cells. J. Nutr. Sci. Vitaminol..

[18] Zhao C., Lai S., Wu D., Liu D., Zou X., Ismail A., El-Seedi H., Arroo R.R., Xiao J. (2020). miRNAs as regulators of antidiabetic effects of fucoidans. eFood.

[19] Luthuli S., Wu S., Cheng Y., Zheng X., Wu M., Tong H. (2019). Therapeutic effects of fucoidan: A review on recent studies. Mar. Drugs.

[20] Lu C., Gu Q., Yu X. (2024). The preparation and anti-atherosclerotic effects of different low-molecular weights fucoidan. Food Biosci..

[21] Bruhn A., Janicek T., Manns D., Nielsen M.M., Balsby T.J.S., Meyer A.S., Rasmussen M.B., Hou X., Saake B., Göke C. (2017). Crude fucoidan content in two North Atlantic kelp species, *Saccharina latissima* and *Laminaria digitata*—Seasonal variation and impact of environmental factors. J. Appl. Phycol..

[22] Zayed A., Ulber R. (2019). Fucoidan production: Approval key challenges and opportunities. Carbohydr. Polym..

[23] Kim S.-K. (2013). Marine Nutraceuticals: Prospects and Perspectives.

[24] Wang Y., Xing M., Cao Q., Ji A., Liang H., Song S. (2019). Biological activities of fucoidan and the factors mediating its therapeutic effects: A review of recent studies. Mar. Drugs.

[25] Silchenko A.S., Rasin A.B., Zueva A.O., Kusaykin M.I., Zvyagintseva T.N., Rubtsov N.K., Ermakova S.P. (2021). Discovery of a fucoidan endo-4O-sulfatase: Regioselective 4O-desulfation of fucoidans and its effect on anticancer activity in vitro. Carbohydr. Polym..

[26] Kasai A., Arafuka S., Koshiba N., Takahashi D., Toshima K. (2015). Systematic synthesis of low-molecular weight fucoidan derivatives and their effect on cancer cells. Org. Biomol. Chem..

[27] Liu M., Liu Y., Cao M.-J., Liu G.-M., Chen Q., Sun L., Chen H. (2017). Antibacterial activity and mechanisms of depolymerized fucoidans isolated from *Laminaria japonica*. Carbohydr. Polym..

[28] Chen Q., Kou L., Wang F., Wang Y. (2019). Size-dependent whitening activity of enzyme-degraded fucoidan from *Laminaria japonica*. Carbohydr. Polym..

[29] Wen Y., Gao L., Zhou H., Ai C., Huang X., Wang M., Zhang Y., Zhao C. (2021). Opportunities and challenges of algal fucoidan for diabetes management. Trends Food Sci. Technol..

[30] Rajauria G., Ravindran R., Garcia-Vaquero M., Rai D.K., Sweeney T., O’Doherty J. (2023). Purification and molecular characterization of fucoidan isolated from *Ascophyllum nodosum* brown seaweed grown in Ireland. Mar. Drugs.

[31] Kusaykin M.I., Silchenko A.S., Zakharenko A.M., Zvyagintseva T.N. (2016). Fucoidanases. Glycobiology.

[32] Sun T., Zhang X., Miao Y., Zhou Y., Shi J., Yan M., Chen A. (2018). Studies on Antiviral and Immuno-Regulation Activity of Low Molecular Weight Fucoidan from *Laminaria japonica*. J. Ocean. Univ. China.

[33] Choi J.-i., Kim H.-J. (2013). Preparation of low molecular weight fucoidan by gamma-irradiation and its anticancer activity. Carbohydr. Polym..

[34] Wang J., Zhang Q., Zhang Z., Song H., Li P. (2010). Potential antioxidant and anticoagulant capacity of low molecular weight fucoidan fractions extracted from *Laminaria japonica*. Int. J. Biol. Macromol..

[35] Park E.J., Choi J.-i. (2017). Melanogenesis inhibitory effect of low molecular weight fucoidan from *Undaria pinnatifida*. J. Appl. Phycol..

[36] Tsai H.-L., Tai C.-J., Huang C.-W., Chang F.-R., Wang J.-Y. (2017). Efficacy of low-molecular-weight fucoidan as a supplemental therapy in metastatic colorectal cancer patients: A double-blind randomized controlled trial. Mar. Drugs.

[37] Qiu Y., Jiang H., Dong Y., Wang Y., Hamouda H.I., Balah M.A., Mao X. (2022). Expression and biochemical characterization of a novel fucoidanase from *Flavobacterium algicola* with the principal product of fucoidan-derived disaccharide. Foods.

[38] Wang Y., Niu D., Que F., Li Y., Chen Q. (2021). Low molecular weight fucoidan prepared by fucoidanase degradation—A promising browning inhibitor. LWT.

[39] Colin S., Deniaud E., Jam M., Descamps V., Chevolot Y., Kervarec N., J.-Yvin C., Barbeyron T., Michel G., Kloareg B. (2006). Cloning and biochemical characterization of the fucanase FcnA: Definition of a novel glycoside hydrolase family specific for sulfated fucans. Glycobiology.

[40] Shen J., Chang Y., Zhang Y., Mei X., Xue C. (2020). Discovery and characterization of an endo-1, 3-fucanase from marine bacterium *Wenyingzhuangia fucanilytica*: A novel glycoside hydrolase family. Front. Microbiology..

[41] Liu G., Shen J., Chang Y., Mei X., Chen G., Zhang Y., Xue C. (2023). Characterization of an endo-1,3-fucanase from marine bacterium *Wenyingzhuangia aestuarii*: The first member of a novel glycoside hydrolase family GH174. Carbohydr. Polym..

[42] Shen J., Zheng L., Zhang Y., Chen G., Mei X., Chang Y., Xue C. (2024). Discovery of a catalytic domain defines a new glycoside hydrolase family containing endo-1,3-fucanase. Carbohydr. Polym..

[43] Vuillemin M., Silchenko A.S., Cao H.T.T., Kokoulin M.S., Trang V.T.D., Holck J., Ermakova S.P., Meyer A.S., Mikkelsen M.D. (2020). Functional characterization of a new GH107 endo-α-(1,4)-fucoidanase from the marine bacterium *Formosa haliotis*. Mar. Drugs.

[44] Trang V.T.D., Mikkelsen M.D., Vuillemin M., Meier S., Cao H.T.T., Muschiol J., Perna V., Nguyen T.T., Tran V.H.N., Holck J. (2022). The endo-α (1,4) specific fucoidanase Fhf2 from *Formosa haliotis* releases highly sulfated fucoidan oligosaccharides. Front. Plant Sci..

[45] Zhu C., Liu Z., Ren L., Jiao S., Zhang X., Wang Q., Li Z., Du Y., Li J.-J. (2021). Overexpression and biochemical characterization of a truncated endo-α (1 → 3)-fucoidanase from *Alteromonas* sp. SN-1009. Food Chem..

[46] Manivasagan P., Oh J. (2015). Production of a novel fucoidanase for the green synthesis of gold nanoparticles by Streptomyces sp. and its cytotoxic effect on HeLa cells. Mar. Drugs.

[47] Gonzalez J.A., Ponce A., Stortz C.A., Lozada M., Dionisi H.M. Mining an Intertidal Sediment Metagenome for Fucanases for the Production of Oligosaccharides from Brown Algae Fucoidans. Red Argentina de Tecnología Enzimática 2021. https://ri.conicet.gov.ar/handle/11336/183000.

[48] Kim W.-J., Kim S.-M., Lee Y.-H., Kim H.-G., Kim H.-K., Moon S.-H., Suh H.-H., Jang K.-H., Park Y.-I. (2008). Isolation and characterization of marine bacterial strain degrading fucoidan from Korean *Undaria pinnatifida* sporophylls. J. Microbiol. Biotechnol..

[49] Setyawan A., Juliasih N.L.G.R., Darmawan M., Susanto G.N., Sarida M. (2023). Screening, characterization, and identification of fucoidanase producing bacteria from *Sargassum polycystum*. AACL Bioflux.

[50] Burtseva Y.V., Kusaikin M., Sova V., Shevchenko N., Skobun A., Zvyagintseva T.N. (2000). Distribution of fucoidan hydrolases and some glycosidases among marine invertebrates. Russ. J. Mar. Biol..

[51] Liu S., Wang Q., Shao Z., Liu Q., He Y., Ren D., Yang H., Li X. (2023). Purification and Characterization of the enzyme fucoidanase from *Cobetia amphilecti* utilizing fucoidan from *Undaria pinnatifida*. Foods.

[52] Tran V.H.N., Perna V., Mikkelsen M.D., Nguyen T.T., Dieu Trang V.T., Baum A., Thuy Cao H.T., Thanh Van T.T., Meyer A.S. (2022). A new FTIR assay for quantitative measurement of endo-fucoidanase activity. Enzym. Microb. Technol..

[53] Furukawa S.-i., Fujikawa T., Koga D., Ide A. (1992). Purification and some properties of exo-type fucoidanases from *Vibrio* sp. N-5. Biosci. Biotechnol. Biochem..

[54] Kim W.J., Park J.W., Park J.K., Choi D.J., Park Y.I. (2015). Purification and characterization of a fucoidanase (FNase S) from a marine bacterium *Sphingomonas paucimobilis* PF-1. Mar. Drugs.

[55] Nagao T., Arai Y., Yamaoka M., Komatsu F., Yagi H., Suzuki H., Ohshiro T. (2018). Identification and characterization of the fucoidanase gene from *Luteolibacter algae* H18. J. Biosci. Bioeng..

[56] Qianqian W., Shuang M., Hourong X., Min Z., Jingmin C. (2011). Purification and the secondary structure of fucoidanase from *Fusarium* sp. LD8. Evid. Based Complement. Altern. Med..

[57] Wu Q., Zhang M., Wu K., Liu B., Cai J., Pan R. (2011). Purification and characteristics of fucoidanase obtained from *Dendryphiella arenaria* TM94. J. Appl. Phycol..

[58] Gomaa M., Fawzy M.A., Hifney A.F., Abdel-Gawad K.M. (2019). Optimization of enzymatic saccharification of fucoidan and alginate from brown seaweed using fucoidanase and alginate lyase from the marine fungus *Dendryphiella arenaria*. J. Appl. Phycol..

[59] Garuba E.O., Adeleye P.A., Onilude A.A. (2020). Purification and properties of thermostable fucoidanase produced by recently isolated terrestrial *Aspergillus flavus* FS018: Characteristics of fucoidanase extracted from *Aspergillus flavus* FS018. Trends Pept. Protein Sci..

[60] Silchenko A.S., Kusaykin M.I., Zakharenko A.M., Menshova R.V., Khanh H.H.N., Dmitrenok P.S., Isakov V.V., Zvyagintseva T.N. (2014). Endo-1,4-fucoidanase from Vietnamese marine mollusk *Lambis* sp. which producing sulphated fucooligosaccharides. J. Mol. Catal. B: Enzym..

[61] Kitamura K., Matsuo M., Tsuneo Y. (1992). Enzymic degradation of fucoidan by fucoidanase from the hepatopancreas of Patinopecten yessoensis. Biosci. Biotechnol. Biochem..

[62] Mikkelsen M.D., Tran V.H.N., Meier S., Nguyen T.T., Holck J., Cao H.T.T., Van T.T.T., Thinh P.D., Meyer A.S., Morth J.P. (2023). Structural and functional characterization of the novel endo-α (1,4)-fucoidanase Mef1 from the marine bacterium *Muricauda eckloniae*. Acta Crystallogr. Sect. D Struct. Biol..

[63] Tran V.H.N., Nguyen T.T., Meier S., Holck J., Cao H.T.T., Van T.T.T., Meyer A.S., Mikkelsen M.D.J. (2022). The endo-α (1, 3)-fucoidanase Mef2 releases uniquely branched oligosaccharides from *Saccharina latissima* fucoidans. Mar. Drugs.

[64] Silchenko A.S., Kusaykin M.I., Kurilenko V.V., Zakharenko A.M., Isakov V.V., Zaporozhets T.S., Gazha A.K., Zvyagintseva T.N.J. (2013). Hydrolysis of fucoidan by fucoidanase isolated from the marine bacterium, *Formosa algae*. Mar. Drugs.

[65] Silchenko A.S., Ustyuzhanina N.E., Kusaykin M.I., Krylov V.B., Shashkov A.S., Dmitrenok A.S., Usoltseva R.V., Zueva A.O., Nifantiev N.E., Zvyagintseva T.N. (2017). Expression and biochemical characterization and substrate specificity of the fucoidanase from *Formosa algae*. Glycobiology.

[66] Zueva A.O., Silchenko A.S., Rasin A.B., Kusaykin M.I., Usoltseva R.V., Kalinovsky A.I., Kurilenko V.V., Zvyagintseva T.N., Thinh P.D., Ermakova S.P. (2020). Expression and biochemical characterization of two recombinant fucoidanases from the marine bacterium *Wenyingzhuangia fucanilytica* CZ1127T. Int. J. Biol. Macromol..

[67] Vickers C., Liu F., Abe K., Salama-Alber O., Jenkins M., Springate C.M.K., Burke J.E., Withers S.G., Boraston A.B. (2018). Endo-fucoidan hydrolases from glycoside hydrolase family 107 (GH107) display structural and mechanistic similarities to fucosidases from GH29. J. Biol. Chem..

[68] Shen J., Chen G., Zhang Y., Mei X., Chang Y., Xue C. (2023). Characterization of a novel endo-1,3-fucanase from marine bacterium *Wenyingzhuangia fucanilytica* reveals the presence of diversity within glycoside hydrolase family 168. Carbohydr. Polym..

[69] Chen G., Dong S., Zhang Y., Shen J., Liu G., Chen F., Li X., Xue C., Cui Q., Feng Y. (2024). Structural investigation of Fun168A unraveling the recognition mechanism of endo-1,3-fucanase towards sulfated fucan. Int. J. Biol. Macromol..

[70] Shen J., Chen G., Zhang Y., Mei X., Zheng L., Xue C., Chang Y. (2024). Characterization of a novel endo-1,3-fucanase from *Wenyingzhuangia fucanilytica* within glycoside hydrolase family 168. Int. J. Biol. Macromol..

[71] Shen J., Liu G., Chen G., Zhang Y., Mei X., Zheng L., Xue C., Chang Y. (2024). Biochemical characterization and cleavage specificities analyses of three endo-1,3-fucanases within glycoside hydrolase family 174. Carbohydr. Polym..

[72] Khanh H.H.N., Trang V.T.D., Thinh P.D., San P.T.J. (2019). Catalytic conditions of fucoidan degrading enzymes from *Vasticardium flavum*. Vietnam. J. Sci. Technol..

[73] Wang L., Wu J., Wang Y., Qin M., Chen Q., Chen T. (2024). Heterologous Expression and Enzymatic Properties of Fucoidanase Fcn1 from Marine *Flavobacterium* sp. J. Food Sci. Technol..

[74] Mohammed A., Guda C. (2011). Computational Approaches for Automated Classification of Enzyme Sequences. J. Proteom. Bioinform..

[75] Kundrotas P.J., Lensink M.F., Alexov E. (2008). Homology-based modeling of 3D structures of protein–protein complexes using alignments of modified sequence profiles. Int. J. Biol. Macromol..

[76] Hospital A., Goñi J.R., Orozco M., Gelpí J.L. (2015). Molecular dynamics simulations: Advances and applications. Adv. Appl. Bioinform. Chem..

[77] Chen Q., Zhang W., Mu W. (2021). Molecular Dynamics Simulation for Food Enzyme Engineering: Why This Technique Should Be Encouraged To Learn. J. Agric. Food Chem..

[78] Mikkelsen M.D., Cao H.T.T., Roret T., Rhein-Knudsen N., Holck J., Tran V.T.T., Nguyen T.T., Tran V.H.N., Lezyk M.J., Muschiol J. (2021). A novel thermostable prokaryotic fucoidan active sulfatase PsFucS1 with an unusual quaternary hexameric structure. Sci. Rep..

[79] Silchenko A.S., Taran I.V., Usoltseva R.V., Zvyagintsev N.V., Zueva A.O., Rubtsov N.K., Lembikova D.E., Nedashkovskaya O.I., Kusaykin M.I., Isaeva M.P.J. (2023). The Discovery of the Fucoidan-Active Endo-1→ 4-α-L-Fucanase of the GH168 Family, Which Produces Fucoidan Derivatives with Regular Sulfation and Anticoagulant Activity. Int. J. Mol. Sci..

[80] Gonzalez J.A., Ponce N.M., Lozada M., Daglio Y., Stortz C.A., Dionisi H.M.J. (2024). Fucanases Related to the GH107 Family from Members of the PVC Superphylum. J. Mar. Sci. Eng..

[81] Liu G., Chang Y., Mei X., Chen G., Zhang Y., Jiang X., Tao W., Xue C. (2024). Identification and structural characterization of a novel chondroitin sulfate-specific carbohydrate-binding module: The first member of a new family, CBM100. Int. J. Biol. Macromol..

[82] Boraston A.B., Bolam D.N., Harry Gilbert J., Davies G.J. (2004). Carbohydrate-binding modules: Fine-tuning polysaccharide recognition. Biochem. J..

[83] Mei X., Chang Y., Shen J., Zhang Y., Chen G., Liu Y., Xue C. (2022). Characterization of a sulfated fucan-specific carbohydrate-binding module: A promising tool for investigating sulfated fucans. Carbohydr. Polym..

[84] Moller I., Sørensen I., Bernal A.J., Blaukopf C., Lee K., Øbro J., Pettolino F., Roberts A., Mikkelsen J.D., Knox J.P.J. (2007). High-throughput mapping of cell-wall polymers within and between plants using novel microarrays. Plant J..

[85] Mei X., Liu G., Shen J., Chen G., Zhang Y., Xue C., Chang Y. (2023). Discovery of a sulfated fucan-specific carbohydrate-binding module: The first member of a new carbohydrate-binding module family. Int. J. Biol. Macromol..

[86] Ivanova E.P., Bakunina I.Y., Nedashkovskaya O.I., Gorshkova N.M., Alexeeva Y.V., Zelepuga E.A., Zvaygintseva T.N., Nicolau D.V., Mikhailov V.V. (2003). Ecophysiological Variabilities in Ectohydrolytic Enzyme Activities of Some *Pseudoalteromonas* Species, *P. citrea*, *P. issachenkonii*, and *P. nigrifaciens*. Curr. Microbiol..

[87] Zhang C., Xue C., Yu L., Wang Y., Xu X., Chang Y. (2013). Fucoidanase activity determination method on basis of pHBH method. J. Chin. Inst. Food Sci. Technol..

[88] Bilan M.I., Kusaykin M.I., Grachev A.A., Tsvetkova E.A., Zvyagintseva T.N., Nifantiev N.E., Usov A.I. (2005). Effect of Enzyme Preparation from the Marine Mollusk *Littorina kurila* on Fucoidan from the Brown Alga *Fucus distichus*. Biochemistry.

[89] Sasaki K., Sakai T., Kojima K., Nakayama S., Nakanishi Y., Kato I.J. (1996). Partial purification and characterization of an enzyme releasing 2-sulfo-α-l-fucopyranose from 2-sulfo-α-l-fucopyranosyl-(1→ 2) pyridylaminated fucose from a sea urchin, *Strongylocentrotus nudus*. Biosci. Biotechnol. Biochem..

[90] Silchenko A.S., Rasin A.B., Kusaykin M.I., Kalinovsky A.I., Miansong Z., Changheng L., Malyarenko O., Zueva A.O., Zvyagintseva T.N., Ermakova S.P. (2017). Structure, enzymatic transformation, anticancer activity of fucoidan and sulphated fucooligosaccharides from *Sargassum horneri*. Carbohydr. Polym..

[91] Arai Y., Shingu Y., Yagi H., Suzuki H., Ohshiro T. (2022). Occurrence of different fucoidanase genes in *Flavobacterium* sp. SW and enzyme characterization. J. Biosci. Bioeng..

[92] Silchenko A.S., Imbs T.I., Zvyagintseva T.N., Fedoreyev S.A., Ermakova S.P. (2017). Brown Alga Metabolites—Inhibitors of Marine Organism Fucoidan Hydrolases. Chem. Nat. Compd..

[93] Imbs T.I., Silchenko A.S., Fedoreev S.A., Isakov V.V., Ermakova S.P., Zvyagintseva T.N. (2018). Fucoidanase inhibitory activity of phlorotannins from brown algae. Algal Res..

[94] Nagae M., Tsuchiya A., Katayama T., Yamamoto K., Wakatsuki S., Kato R. (2007). Structural Basis of the Catalytic Reaction Mechanism of Novel 1,2-L-Fucosidase from *Bifidobacterium bifidum*. J. Biol. Chem..

[95] Davies G.J., Wilson K.S., Henrissat B.J. (1997). Nomenclature for sugar-binding subsites in glycosyl hydrolases. Biochem. J..

[96] Zueva A.O., Silchenko A.S., Rasin A.B., Malyarenko O.S., Kusaykin M.I., Kalinovsky A.I., Ermakova S.P. (2023). Production of high- and low-molecular weight fucoidan fragments with defined sulfation patterns and heightened in vitro anticancer activity against TNBC cells using novel endo-fucanases of the GH107 family. Carbohydr. Polym..

[97] Xu X., Chang Y., Xue C., Wang J., Shen J. (2018). Gastric Protective Activities of Sea Cucumber Fucoidans with Different Molecular Weight and Chain Conformations: A Structure–Activity Relationship Investigation. J. Agric. Food Chem..

[98] Silchenko A.S., Rasin A.B., Kusaykin M.I., Malyarenko O.S., Shevchenko N.M., Zueva A.O., Kalinovsky A.I., Zvyagintseva T.N., Ermakova S.P. (2018). Modification of native fucoidan from Fucus evanescens by recombinant fucoidanase from marine bacteria *Formosa algae*. Carbohydr. Polym..

[99] Ptak S.H., Hjuler A.L., Ditlevsen S.I., Fretté X., Errico M., Christensen K.V. (2021). The effect of seasonality and geographic location on sulphated polysaccharides from brown algae. Aquac. Res..

[100] Honya M., Mori H., Anzai M., Araki Y., Nisizawa K., Kain J.M., Brown M.T., Lahaye M. (1999). Monthly Changes in the Content of fucans, Their Constituent Sugars and Sulphate in Cultured Laminaria Japonica, Sixteenth Interna-tional Seaweed Symposium, Dordrecht, 1999.

[101] Fletcher H.R., Biller P., Ross A.B., Adams J.M.M. (2017). The seasonal variation of fucoidan within three species of brown macroalgae. Algal Res..

[102] Hifney A.F., Gomaa M., Fawzy M.A., Abdel-Gawad K.M. (2019). Optimizing a Low-Cost Production Process of Crude Fucoidanase by *Dendryphiella arenaria* Utilizing *Cystoseira trinodis* (Phaeophyceae) and Enzymatic Hydrolysis of the Brown Algal Biomass. Waste Biomass Valorization.

[103] Ohmes J., Mikkelsen M.D., Nguyen T.T., Tran V.H.N., Meier S., Nielsen M.S., Ding M., Seekamp A., Meyer A.S., Fuchs S. (2022). Depolymerization of fucoidan with endo-fucoidanase changes bioactivity in processes relevant for bone regeneration. Carbohydr. Polym..

[104] Chen G., Yu L., Zhang Y., Chang Y., Liu Y., Shen J., Xue C. (2021). Utilizing heterologously overexpressed endo-1,3-fucanase to investigate the structure of sulfated fucan from sea cucumber (*Holothuria hilla*). Carbohydr. Polym..

